# Tailored multifunctional polysiloxanes as advanced emulsifiers in colloid systems

**DOI:** 10.1038/s41598-025-17552-7

**Published:** 2025-09-29

**Authors:** Anna Olejnik, Bogna Sztorch, Miłosz Frydrych, Klaudia Krysiak-Smułek, Julia Leśniewska, Robert E. Przekop

**Affiliations:** 1 Center for Advanced Technologies, Adam Mickiewicz University, Uniwersytetu Poznańskiego 10, 61-614 Poznań, Poland; 2 Present Address: Faculty of Chemistry, Adam Mickiewicz University, Uniwersytetu Poznańskiego 8, 61-614 Poznań, Poland

**Keywords:** Polysiloxanes, Emulsifiers, Colloidal stability, Hydrosilylation, Interfacial engineering, Multifunctional polysiloxanes, Chemistry, Materials science

## Abstract

This study investigates the synthesis, physicochemical characterization, and emulsifying performance of multifunctional polysiloxane modified with trimethoxysilane, eugenol and octane in varying molar ratios. The functionalized organosilicon compounds were synthesized by hydrosilylation and characterized using NMR, FT-IR, thermogravimetric analysis, and contact angle measurements. Their emulsifying properties were evaluated in oil-water emulsions, with stability assessed through centrifugation tests, multiple light scattering, and optical microscopy. The polysiloxane modified with trimethoxysilane: eugenol: octane with a 1:4:3 molar ratio exhibited the highest thermal stability. Emulsions formulated with this compound demonstrated superior physical stability, with backscattering destabilization rates as low as − 0.29%/day, attributed to enhanced interfacial interactions and hydrogen bonding. Emulsion containing polysiloxane modified only with eugenol and octane exhibited the lowest stability, with early phase separation observed after 1.5 h and backscattering rates reaching − 1.06%/day. These results highlight the critical role of silane functionalities in interfacial stabilization. These findings demonstrate the potential of structurally tailored polysiloxanes as advanced emulsifiers for applications in cosmetics, pharmaceuticals, and other industries.

Polysiloxanes are organosilicon compounds with chain consisting of alternating silicon and oxygen atoms. Due to their remarkable properties, such as high chemical and thermal stability, biocompatibility, moisture resistance, film-forming ability, and low cost, they have found applications in various fields including electronics, automotive, construction, personal care, and healthcare^1^. They have been used in organic light-emitting diodes (OLEDs), solar cells, electrical memories, and liquid crystalline materials^2^. In medical field, polysiloxanes are applied in artificial organs, prostheses, facial reconstruction devices, tubes, and catheters^3^. Functionalized polysiloxanes are also utilized in the textile industry^4^. In recent years, they have been increasingly applied in daily use and beauty products as ingredients in cosmetic formulations, acting as emollients, viscosity regulators, or foam stabilizers^5,6^. Various chemical groups can be attached to polysiloxane core in catalytic hydrosilylation, an effective method for producing a wide range of derivatives^7^. To date, polysiloxanes have been functionalized with perylene diimides and poly(ethylene glycol)^7^phosphazene^8^*N*-allylaniline, *N*-allylcyclohexylamine, 4-vinylpyridine^9^aminoalkyl^10^ and fluoroalkyl groups^11^. Such modifications with organic substituents enable the design of amphiphilic compounds that contain both a hydrophilic and a hydrophobic part. In these systems, the hydrophobic alkyl units interact with the oil phase, hydrophilic chains associate to the aqueous phase, while the siloxane backbone localizes at the phase boundary providing structural stability.

Additionally, multifunctional polysiloxanes, due to their unique physicochemical properties, have found applications in the modification of materials with specialized functionalities. For instance, one study demonstrated that the incorporation of polysiloxane-based modifiers into an epoxy gelcoat significantly enhanced its anti-icing properties, reducing ice adhesion by approximately 50% compared to the unmodified material^12^. Other studies have shown that the addition of multifunctional polysiloxanes can improve the mechanical performance of thermoplastics, such as increasing impact strength and elongation at break in PLA-based composites^13^. Similarly, in FDM-printed ABS composites, the use of polysiloxane additives improved both silica dispersion and mechanical properties^14^.

Polysiloxanes are also employed in a wide range of other applications, including specialty adhesives with antibacterial properties^15^, surfactants^16^, sorbents^17^, and as resin modifiers^18,19^. Due to their newly designed structure, the multifunctional polysiloxanes may affect the stability of the emulsion. This aspect was discussed by Gruning et al., who designed an organomodified silicone copolymer, which turned out to be a better emulsifier than triglycerine trioleate^20^.

Based on these findings, this study aimed to obtain and characterize new poly(methylhydrogen) siloxane derivatives that will act as an emulsifier in colloidal systems. To achieve this goal, three substituents such as octane, trimethoxysilane, and eugenol were introduced in varying molar ratios to change the nature of polysiloxanes. Additionally, modification of organosilicon compounds with eugenol may contribute to obtaining compounds with potential antioxidant and antibacterial properties^21,22^. The novelty of our study lies in the preparation of multifunctional siloxanes as a new class of compounds with potential applications as emulsifiers, foam stabilizer, in textile softeners. By introducing different functional groups in various molar ratios along the siloxane chain, it is possible to obtain a wide range of structural combinations, which opens opportunities for the design of new types of emulsifiers. Importantly, the use of a one-pot synthesis significantly simplifies the preparation process, making it more cost-effective compared to other approaches based on functionalized hydrocarbons. Furthermore, the conversion of these reactions is very high with no by-products formed, which highlights the efficiency of the proposed method.

## Materials and methods

### Synthesis of modified polysiloxanes

**Fig. 1:**
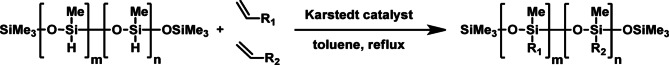
Synthesis scheme of modified polysiloxanes.

The modified polysiloxanes (PWS) were obtained in hydrosilylation reaction using commercially available olefins. The general scheme of the reaction is presented in Fig. 1. The substrates used to conduct the reaction are collected in Table 1.

**Table 1:** Substrates applied in in hydrosilylation reaction.

Sample name	Structure	Core	Olefin 1	Olefin 2	Olefin 3	Molar ratio
PWS1		PWS991	VTMS	EUG	OCT	1:4:3
PWS2		PWS991	VTMS	EUG	OCT	1:2:1
PWS3		PWS991	-	EUG	OCT	3:1

*VTMS- vinyltrimethoxysilane, EUG- eugenol, OCT- octene.

In an optimizing procedure, 30 g of PWS and a mixture of:

PWS1 - vinyltrimethoxysilane (9.64 g), eugenol (42.73 g) and octene (21.90 g) in molar ratio 1:4:3,

PWS2 - vinyltrimethoxysilane (19.29 g), eugenol (42.73 g) and octene (14.60 g) in molar ratio 1:2:1,

PWS3 - eugenol (64.10 g) and octene (14.60 g) in molar ratio 3:1,

were placed in a three-neck, round-bottom flask, then 250 mL of toluene was added. The reaction mixture was constantly stirred and heated to 60 °C (then 30 µL of Karstedt’s catalyst solution in xylene was added). The process was carried out for 24–48 h under reflux. The progress of the reaction was monitored using FT-IR-ATR spectroscopy (Nicolet iS50, Thermo Scientific, Waltham, Massachusetts, USA) until the Si-H bands’ disappearance (at 2141 cm^− 1^ and 889 cm^− 1^ - stretching and bending vibrations, respectively).

### Characterization of compounds

^1^H^13^, C, and^29^Si Nuclear Magnetic Resonance spectra were recorded on Bruker Ascend 400 and Ultra Shield 300 spectrometers (Bruker Corporation, Billerica, Massachusetts, USA) at 25 °C in CDCl_3_. Chemical shifts are reported in ppm concerning the residual solvent (CHCl_3_) peaks for^1^H and^13^C and^29^Si.

FT-IR spectra were recorded on a Nicolet iS 50 Fourier transform spectrophotometer (Thermo Fisher Scientific, Waltham, Massachusetts, USA) equipped with a diamond ATR unit with a resolution of 0.09 cm^− 1^.

Thermogravimetry (TG, DTG) was performed using a TG 209 F1 Libra (Netzsch, Selb, Germany) gravimetric analyzer. Samples of 5 ± 0.2 mg were cut from each granulate and placed in Al_2_O_3_ crucibles. Measurements were conducted under nitrogen (flow of 20 mL/min) in the 30 ÷ 800 °C range and a 10 °C/min heating rate.

The Water Contact Angle (WCA) measurement was performed by the sessile drop technique at room temperature and atmospheric pressure with a DSA100 goniometer (Krüss Scientific, Hamburg, Germany). Three independent measurements were taken for each sample, each with a 5 µL water drop, and the obtained results were averaged. Samples for measurement were prepared using the dip-coating method. A 5% modifier solution in isopropanol was prepared, and a microscope slide was submerged. Once the modifier had been drip-dried, the measurement of hydrophilic-hydrophobic properties was performed.

The refractive index of multifunctional polysiloxane was determined using a refractometer (InsMark Instrument Technology Co., Ltd, Shanghai, China).

#### Preparation of emulsions

The selected emulsifier (PWS1/PWS2/PWS3) was dissolved in isopropyl myristate, and the solution obtained was stirred for one minute at room temperature using the disperser Ultra Turrax Turbe Drive (IKA, Staufen, Germany). Next, the distilled water was added, and the mixture was stirred at 6000 rpm for one minute. The composition of emulsions is presented in Table 2. Afterwards, the mixture was homogenized at RT for 4 min using the homogenizer T 18 digital Ultra Turrax (IKA, Staufen, Germany) operating at 16 000 rpm.

**Table 2:** The composition of emulsions.

Emulsion	Quantity (% w/w)		
	Emulsifier	Isopropyl myristate	Water
PWS1-E1 PWS2-E1 PWS3-E1	2	48	50
PWS1-E2 PWS2-E2 PWS3-E2	2	28	70
PWS1-E3 PWS2-E3 PWS2-E3	2	18	80

## Characterization of formulations

### Emulsion stability

5 mL of the emulsion was poured into centrifuge tubes. The emulsion was centrifuged at 3000 rpm for 5 min by laboratory MPW-56 centrifuge (MPW Med. Instruments, Warsaw, Poland). Afterward, the total height of the sample and the height of the emulsion were measured. Next, emulsion stability (ES) was calculated by dividing the height of the emulsion by the total height of the sample and multiplying by 100%.

### Stability test of emulsions by multiple light scattering

The emulsion was introduced into a glass flask and was analysed using Multiscan MS 20 (DataPhysics Instruments GmbH, Filderstadt, Germany). Measurements were conducted for 31 days at 25 °C. The results were presented as graphs of transmitted and backscattered light versus sample height. Additionally, based on the data obtained, the plots of backscattered light and stability indexes over time were determined.

### Analysis of droplet size by optical microscope

Analysis was performed using a Digital Light Microscope VHX 7000 (Keyence, Osaka, Japan) with a 100× to 1000× VH-Z100T lens. All the pictures were recorded with a VHX 7020 camera. A small amount of sample was put on the microscope slide and covered with a microscope cover glass. The slide was put on the microscope stage. The position of the stage, lens zoom, and microscope settings were adjusted to visualize the drops of emulsions.

### Statistical analysis

Statistical analysis was conducted to evaluate differences in emulsions’ stability performed by centrifugation test. Each emulsifier-emulsion combination (e.g., PWS1-E1) was treated as an independent group. Pairwise comparison between groups were performed using Welch’s t-test. The level of statistical significance was set at α = 0.05. Significance was interpreted as follows:

*p* < 0.05 significant (*).

*p* < 0.01 highly significant (**).

*p* < 0.001 very highly significant (***).

A one-way ANOVA was conducted separately for each emulsion (E1, E2, E3) to assess whether breakdown rates (% backscattering/day) differed significantly among the three emulsifiers (PWS1, PWS2, PWS3). When significant effect was found (*p* < 0.05), a Tukey’s Honest Significant Difference (HSD) post-hoc test was applied to identify pairwise differences.

## Results and discussion

### Characterization of synthesized compounds

**Fig. 2:**
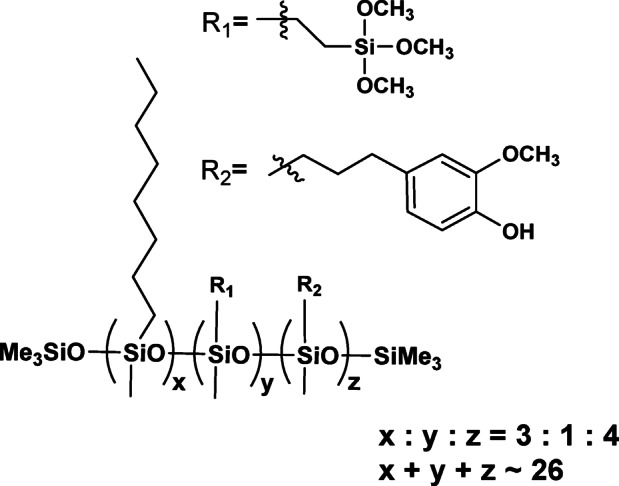
The chemical structure of PWS1.

**Fig. 3:**
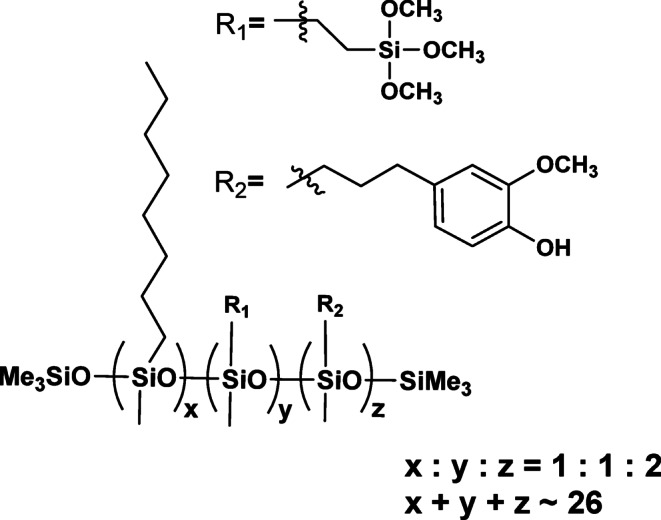
The chemical structure of PWS2.

**Fig. 4:**
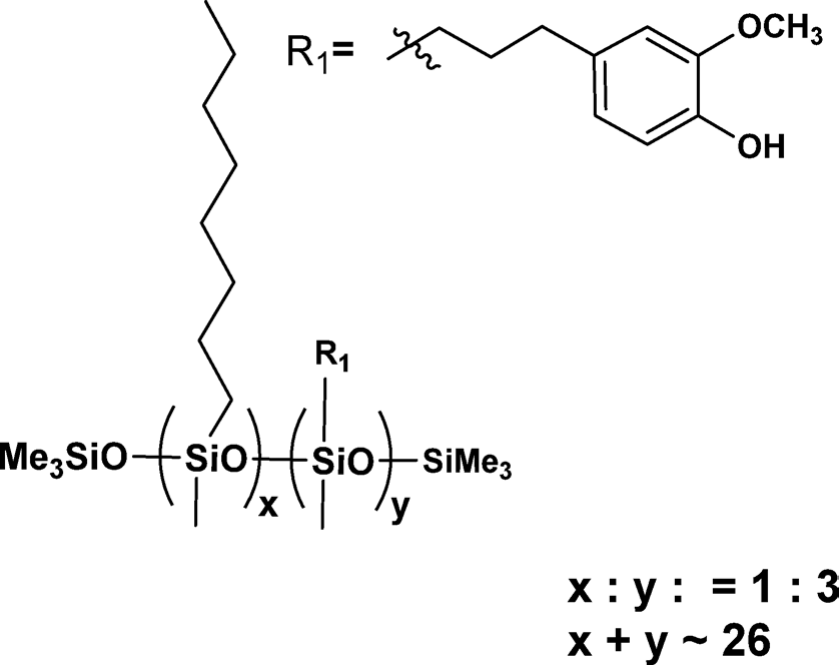
The chemical structure of PWS3.

From a structural perspective, the reaction products constitute a mixture of isomers, as the substitution process lacks selectivity. This has significant implications for the interpretation of their physicochemical and functional properties, which reflect t a complex superposition of both molecular characteristics and intra- and intermolecular interactions. Owing to the linear architecture of the substituted core and the high flexibility of the Si–O–Si bonds, the products can interact with functional groups within the same chain as well as with neighbouring chains. At the current stage of analytical technique development, the quantitative analysis of such mixtures remains a separate research challenge that lies beyond the scope of this study. The chemical structures of the modified polysiloxane obtained are presented in Figs. 2, 3 and 4.

The NMR signals for each compound are shown below:

### NMR analysis of PWS1

^1^H NMR (600 MHz, CDCl_3_) δ (ppm): 6.88–6.63 (m, CH_2_-Ph), 5,67–5.47 (m, O–H), 3.90–3.72 (m, Ph-O-CH_3_), 3.55 (m, Si–O-CH_3_), 2.53 (m, CH_2_-Ph), 1.88 (d, Si-(CH_3_-)CH-CH_2_-Ph) 1.63 (m, CH_2_-CH_2_-Ph), 1.27 (m, Si-CH_2_-(CH_2_)_6_-CH_3_), 0.89 (m, Si-CH_2_-(CH_2_)_6_-CH_3_) 0.54 (m, Si-CH_2_-CH_2_-Si, Si-CH_2_-CH_2_-CH_2_), 0.19–0.0 (m, SiMe_2_).

^13^C NMR (151 MHz, CDCl_3_) δ (ppm): 146.24, 143.54, 137.79, 134.38, 130.67, 130.56, 128.96, 128.15, 125.22, 123.33, 120.89, 120.84, 119.23, 114.29, 114.09, 110.92, 107.79, 55.74, 55.62 (Ph-O-CH_3_), 50.42 (Si-(OMe)_3_), 39.12, 33.41, 31.90, 29.39, 29.31, 25.26, 23.02, 22.63, 21.38, 18.27, 17.61, 17.32, 14.04, 1.80, -0.36 (SiMe_2_).

^29^Si NMR (119 MHz, CDCl_3_) δ (ppm): -21,61- (-22,40) (SiMe, SiMe_3_), -41.62 (Si(OMe)_3_.

### NMR analysis of PWS2

^1^H NMR (600 MHz, CDCl_3_) δ (ppm): 6.88–6.64 (m, CH_2_-Ph), 5,71–5.46 (m, O–H), 3.90–3.75 (m, Ph-O-CH_3_), 3.55 (m, Si–O-CH_3_), 2.53 (m, CH_2_-Ph), 1.88 (d, Si-(CH_3_-)CH-CH_2_-Ph) 1.63 (m, CH_2_-CH_2_-Ph), 1.27 (m, Si-CH_2_-(CH_2_)_6_-CH_3_), 0.89 (m, Si-CH_2_-(CH_2_)_6_-CH_3_) 0.54 (m, Si-CH_2_-CH_2_-Si, Si-CH_2_-CH_2_-CH_2_), 0.19–0.0 (m, SiMe_2_).

^13^C NMR (151 MHz, CDCl_3_) δ (ppm): 146.26, 143.54, 137.79, 134.33, 130.66, 130.56, 128.96, 128.15, 125.22, 123.34, 120.83, 119.23, 114.26, 114.10, 110.94, 107.78, 55.75, 55.63 (Ph-O-CH_3_), 50.43(Si-(OMe)_3_) 39.13, 33.42, 31.90, 29.32, 25.28, 23.03, 22.63, 21.39, 18.27, 17.62, 17.33, 14.04, 13.73, 8.26, 1.80, 0.54, -0.35, -1.31 (SiMe_2_).

^29^Si NMR (119 MHz, CDCl_3_) δ (ppm): -22,10- (-22,66) (SiMe, SiMe_3_), -41.75 (Si(OMe)_3_.

### NMR analysis of PWS3

^1^H NMR (600 MHz, CDCl_3_) δ (ppm): 6.88–6.57 (m, CH_2_-Ph), 5,58–5.48 (m, O–H), 3.90–3.75 (m, Ph-O-CH_3_), 2.52 (m, CH_2_-Ph), 1.88 (d, Si-(CH_3_-)CH-CH_2_-Ph) 1.62 (m, CH_2_-CH_2_-Ph), 1.27 (m, Si-CH_2_-(CH_2_)_6_-CH_3_), 0.89 (m, Si-CH_2_-(CH_2_)_6_-CH_3_) 0.53 (m, Si-CH_2_-CH_2_-CH_2_), 0.15–0.0 (m, SiMe_2_).

^13^C NMR (151 MHz, CDCl_3_) δ (ppm): 146.46, 146.23, 144.67, 143.53, 137.79, 134.28, 130.65, 130.56, 128.96, 128.15, 125.22, 123.33, 120.88, 120.81, 119.23, 114.26, 114.09, 110.93, 107.78, 55.74, 55.63 (Ph-O-CH_3_), 39.10, 33.39, 31.90, 29.31, 25.24, 23.02, 22.64, 21.38, 18.27, 17.62, 17.32, 14.06, 1.82, -0.30, -0.60 (SiMe_2_).

**Fig. 5:**
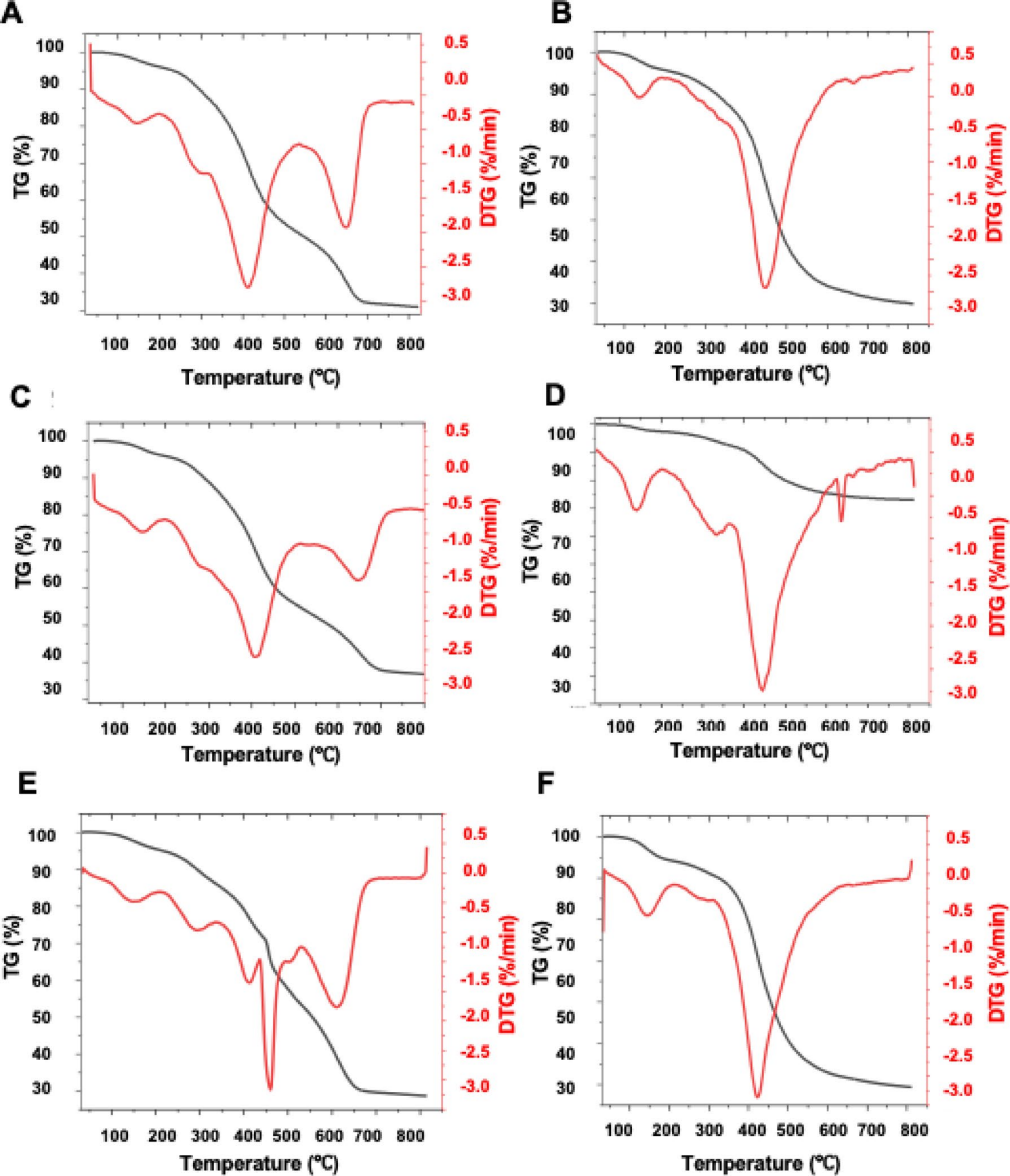
TG and DTG of PWS1 in air (**A**), PWS1 in nitrogen (**B**), PWS2 in air (**C**), PWS2 in nitrogen (**D**), PWS3 in air (**E**), PWS3 in nitrogen (**F**).

^29^Si NMR (119 MHz, CDCl_3_) δ (ppm): -21,20- (-22,94) (SiMe, SiMe_3_),

Thermogravimetric analysis (TGA) is a crucial technique for evaluating the thermal stability of chemical compounds. It provides valuable information on degradation temperatures, oxidation behaviour, and mass-loss processes, all of which are essential for understanding the thermal behaviour of materials. In the case of pharmaceutical or cosmetic raw materials, TGA is particularly important for assessing their stability and durability under processing conditions involved in topical formulation, such as heating, homogenization, and storage. This information is critical for selecting suitable ingredients that ensure the quality, safety, and performance of the final product. The thermal decomposition of the obtained multifunctional compounds proceeds in a characteristic multistep mechanism, which can be attributed to their complex molecular architecture (Fig. 5).

**Table 3:** Results of thermogravimetric analysis in nitrogen.

Sample	5% Mass loss (°C)	Onset temperature (°C)		Temperature at the maximum rate of mass loss (°C) T_max_		Residual mass (%)
		I	II	I	II	
PWS1	230.4	105.3	396.0	136.9	446.4	39.99
PWS2	196.2	111.5	410.2	137.9	444.9	45.80
PWS3	177.2	113.1	380.1	144.4	422.3	39.49

**Table 4:** Results of thermogravimetric analysis in air.

Sample	5% Mass loss (°C)	Onset temperature (°C)			Temperature at the maximum rate of mass loss (°C) T_max_			Residual mass (%)
		I	II	III	I	II	III	
PWS1	235.4	126.6	339.4	610.4	145.1	413.1	648.9	30.81
PWS2	229.4	123.2	338.3	607.4	147.5	406.2	647.1	36.64
PWS3	216.7	255.7	428.5	572.6	275.6	457.0	610.1	28.74

The results of thermogravimetric analysis in nitrogen and air were presented in Tables 3 and 4. Owing to their relatively high molecular weight, the degradation process proceeds with a gradual release of low-molecular-weight volatile products. From the perspective of thermal stability, the susceptibility of the functional groups bonded to the polymer backbone can be ranked in the following order: eugenol moieties < octyl groups < methyl groups < trimethoxysilyl groups. Under oxidative conditions (air atmosphere), a distinct mass loss above 500 °C, corresponding to the secondary decomposition of previously degraded fragments and the oxidation combustion of carbonaceous residues, as confirmed by the evolution of CO₂. In contrast, under an inert (nitrogen) atmosphere, the decomposition pathway favours the formation of a carbon-rich residue, resulting in a comparatively higher residual mass post-pyrolysis. Notably, in the compound with the highest relative content of methylsilyl groups, a distinct, sharp peak appears at approximately 650 °C. This feature is attributed to the thermolysis of these groups, underscoring their distinct contribution to the overall thermal degradation profile of the system.

**Fig. 6:**
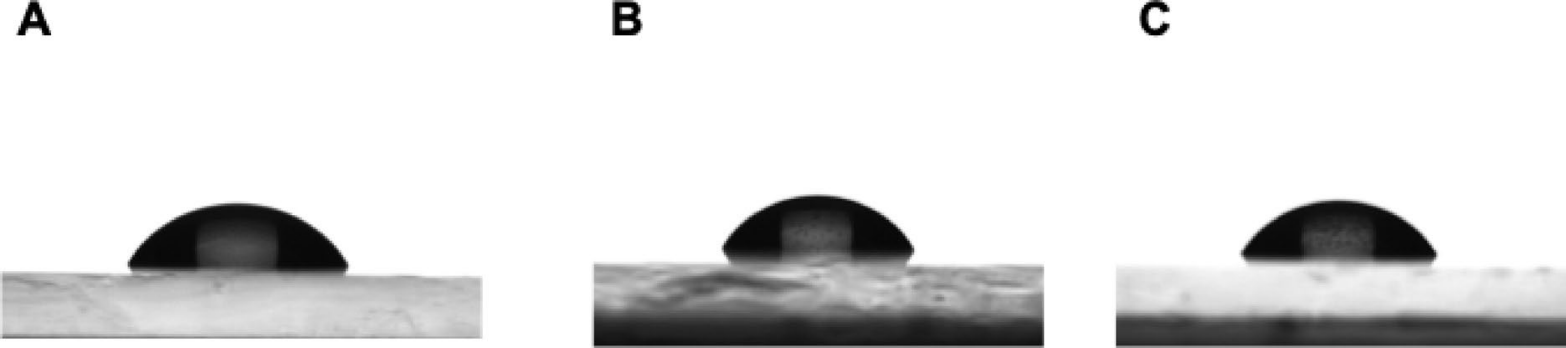
Analysis of water contact angle for PWS1, PWS2, PWS3.

Table 5; Fig. 6 show the results of the water contact angle (WCA) measurements. Surface modification of the glass slide using the dip-coating method led to an increase in contact angle compared to the reference sample (40.04°) and a decrease relative to the unmodified PWS material (81.10°). However, all measured angles fall within the range characteristic of hydrophilic surfaces (< 90°), as expected due to the types of precursors in the synthesis of the modifiers, such as vinyltrimethoxysilane and eugenol. Due to the absence of the trimethoxysilane group in the PWS3 compound, the water contact angle increased.

The refractive indices of polysiloxane samples modified with trimethoxysilane (TMS), eugenol (EUG), and octane (OCT) at different molar ratios reveal distinct structural differences. PWS1 (TMS: EUG: OCT = 1:4:3) showed an intermediate refractive index of 1.4963, reflecting a balance between polar eugenol and hydrophobic octane. PWS2 (1:2:1) had the lowest refractive index of 1.4958, likely due to its lower content of polar and hydrophobic modifiers, resulting in a less dense structure. PWS3 (EUG: OCT = 1:3), lacking TMS, exhibited the highest refractive index of 1.5110, attributed to the absence of siloxane network formation and a dominance of densely packed organic groups. These findings demonstrate that the refractive index and related material properties can be tuned by adjusting the composition, impacting the optical and structural characteristics of the polysiloxane systems.

**Table 5:** Characterization of multifunctional polysiloxanes.

Sample	Contact angle (°)	Refractive index
PWS1	64.3 ± 0.46	1.4963 ± 0.0002
PWS2	66.4 ± 0.90	1.4958 ± 0.0004
PWS3	78.6 ± 1.79	1.5110 ± 0.0009

### Characterization of emulsions

#### Stability studies of emulsions by centrifugation test and multiple light scattering

Emulsion stability refers to the ability of a colloidal system to maintain its particle size distribution over time and under storage conditions^23^. The centrifugation test was performed to evaluate the stability of emulsions under applied centrifugal forces, simulating the effects of long-term gravitational separation. This approach accelerates the separation process in colloidal systems to predict their long-term stability within a shorter time frame. A lower degree of separation corresponds to higher stability of the colloidal system^24^.

**Fig. 7:**
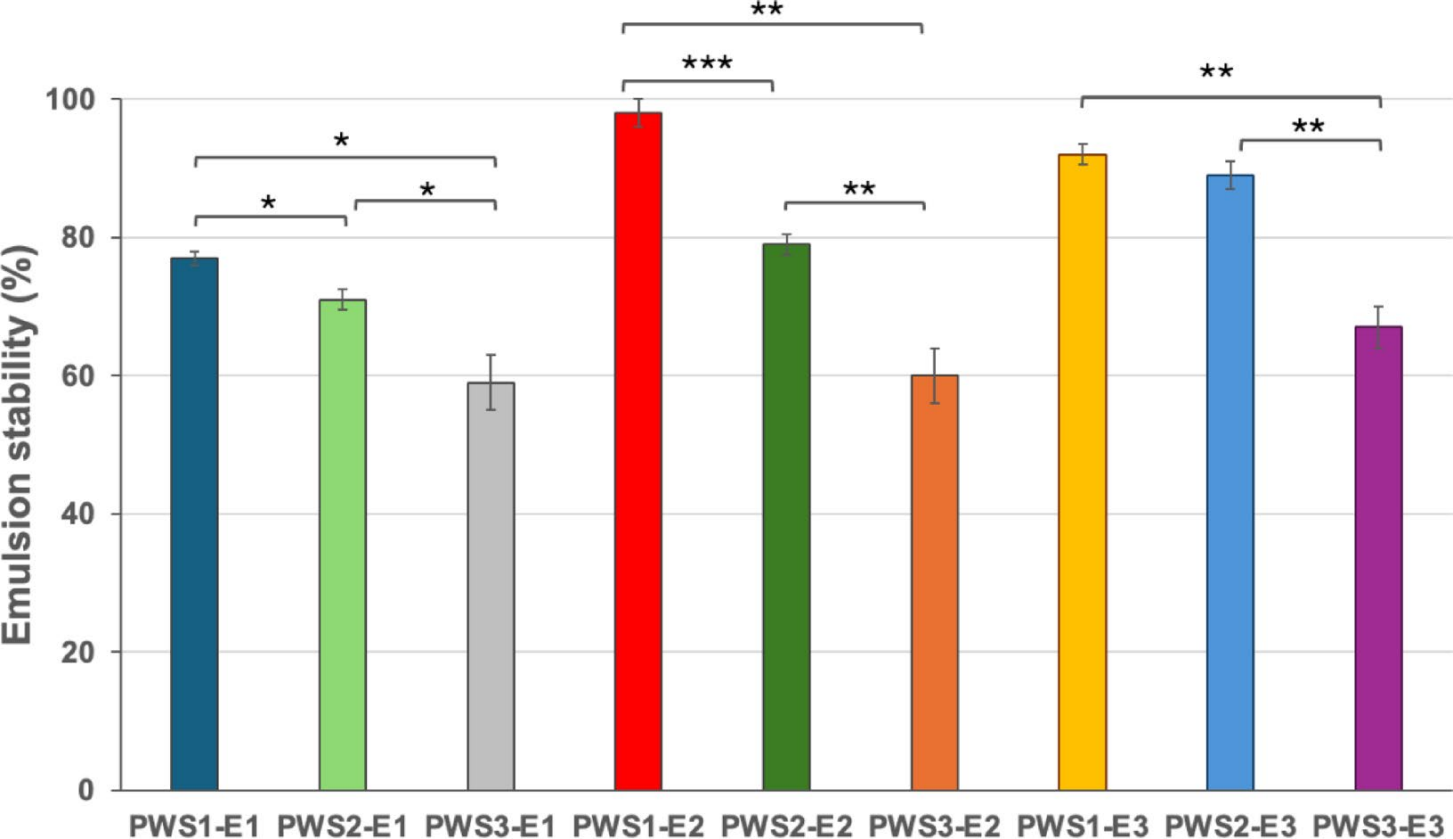
Emulsion stability by centrifugation test.

It was observed that the stability of emulsions varied significantly depending on both the emulsifier type and the water-to-oil ratio used in the formulations (Fig. 7). Among the E1 formulations, PWS1-E1 exhibited significantly higher emulsion stability compared to both PWS2-E1 (*p < 0.05) and PWS3-E1 (*p < 0.05), with PWS3-E1 showing the lowest stability. Similarly, for the E2 formulations, PWS1-E2 displayed superior stability compared to both PWS2-E2 (***p < 0.001) and PWS3-E2 (**p < 0.01), while PWS3-E2 had the poorest performance. Furthermore, PWS1-E2 also showed significantly greater stability than PWS3-E1 (**p < 0.01), indicating the influence of both emulsifier composition and aqueous phase proportion. In the E3 emulsions, PWS1-E3 and PWS2-E3 were both significantly more stable than PWS3-E3 (**p < 0.01 for both comparisons), whereas no significant difference was observed between PWS1-E3 and PWS2-E3 (*p* > 0.05). These findings collectively suggests that PWS1 consistently provided higher emulsion stability across all water-to-oil ratios. This polysiloxane was obtained using as a substrates vinyltrimethoxysilane, eugenol and octene in molar ratio 1:4:3.

It is worth noting that vinyl trimethoxysilane (VTMS) is commonly applied as an adhesion promoter, as well as coupling and crosslinking agent^25^. PWS1 was first dissolved in oil (isopropyl myristate), and afterwards water was added in varying concentrations (50, 70, and 80%w/w). Although TMS groups did not undergo hydrolysis in our experimental conditions, the electronegative oxygen atoms in the Si-O-CH₃ moieties, together with the octane and eugenol substituents, are likely to contribute to the amphiphilic character of PWS1. The stabilization of the emulsions observed in this study can be attributed to the adsorption of the newly synthesized poly(methylhydrogen) siloxane derivatives at the oil–water interface. The incorporation of three distinct substituents—octane, trimethoxysilane, and eugenol—provides complementary contributions to the interfacial properties of these compounds. The hydrophobic octyl groups are proposed to anchor effectively into the oil phase, thereby lowering the interfacial free energy and promoting strong adsorption. The trimethoxysilane substituents, while not undergoing hydrolysis at the experimental conditions (pH ≈ 5.5), nevertheless alter the overall polarity of the polymer compared to unmodified polysiloxane. Furthermore, the eugenol groups provide an additional amphiphilic contribution. Their aromatic ring enhances hydrophobic interactions with the oil phase, whereas the hydroxyl functionality allows for polar interactions and the formation of hydrogen bonding with water, further enhancing interfacial stabilization. The amphiphilicity of PWS1 enhances interfacial packing and adsorption strength, in line with the effects described for functional siloxane surfactants^26^. Taken together, these three functional groups are likely to act synergistically to promote the formation of persistent interfacial film around dispersed droplets. Such a film would act as a barrier to droplet coalescence and slow down Ostwald ripening, thereby explaining the improved stability of the emulsions. In simple terms, the balanced amphiphilicity of PWS1 to enables its effective alignment at the oil-water interface, reducing interfacial tension and stabilizing droplet formation.

In contrast, lower emulsion stability was observed when PWS2 was applied as an emulsifying agent in both emulsion systems (E1, E2). This polysiloxane was functionalized with vinyltrimethoxysilane, eugenol, and octene as substrates in a molar ratio 1:2:1. The lower proportion of eugenol and octyl groups attached to the polysiloxane chain likely resulted in fewer hydrogen bonding interactions, which adversely affected the stability of emulsions. Notably, when the polysiloxane was functionalized solely with eugenol and octane, without the incorporation of TMS groups, the resulting emulsions exhibited the lowest stability compared to other modified polysiloxanes, suggesting a significant reduction in emulsifying properties of PWS3. The lowest stability of PWS3-based emulsions (lacking TMS) supports the critical role of trimethoxysilane groups in interfacial stabilization. PWS3 relied solely on eugenol (weakly polar) and octyl groups (hydrophobic), resulting in poorer emulsification performance. The intermediate water contact angle of PWS1 (64.3°) compared to PWS3 (78.6°) demonstrates that the presence of intact trimethoxysilane (TMS) groups—despite their lack of hydrolysis—introduces sufficient polarity to the polysiloxane structure, thereby optimizing its amphiphilic character for effective interfacial stabilization in emulsion systems.

The stability of the emulsion obtained was also assessed by multiple light scattering. This optical method enables the monitoring of changes in samples without dilution and shortens the duration of aging tests for new formulations^27,28^. During analysis, the sample is scanned with a near-infrared pulsed light source, while two synchronized detectors simultaneously record transmission and backscattering intensity data. This process generates an intensity profile as a function of sample height, providing a macroscopic fingerprint of the system at a given time point^29^. Compared with traditional methods, this technique significantly reduces the time required for stability testing^30^.

**Fig. 8:**
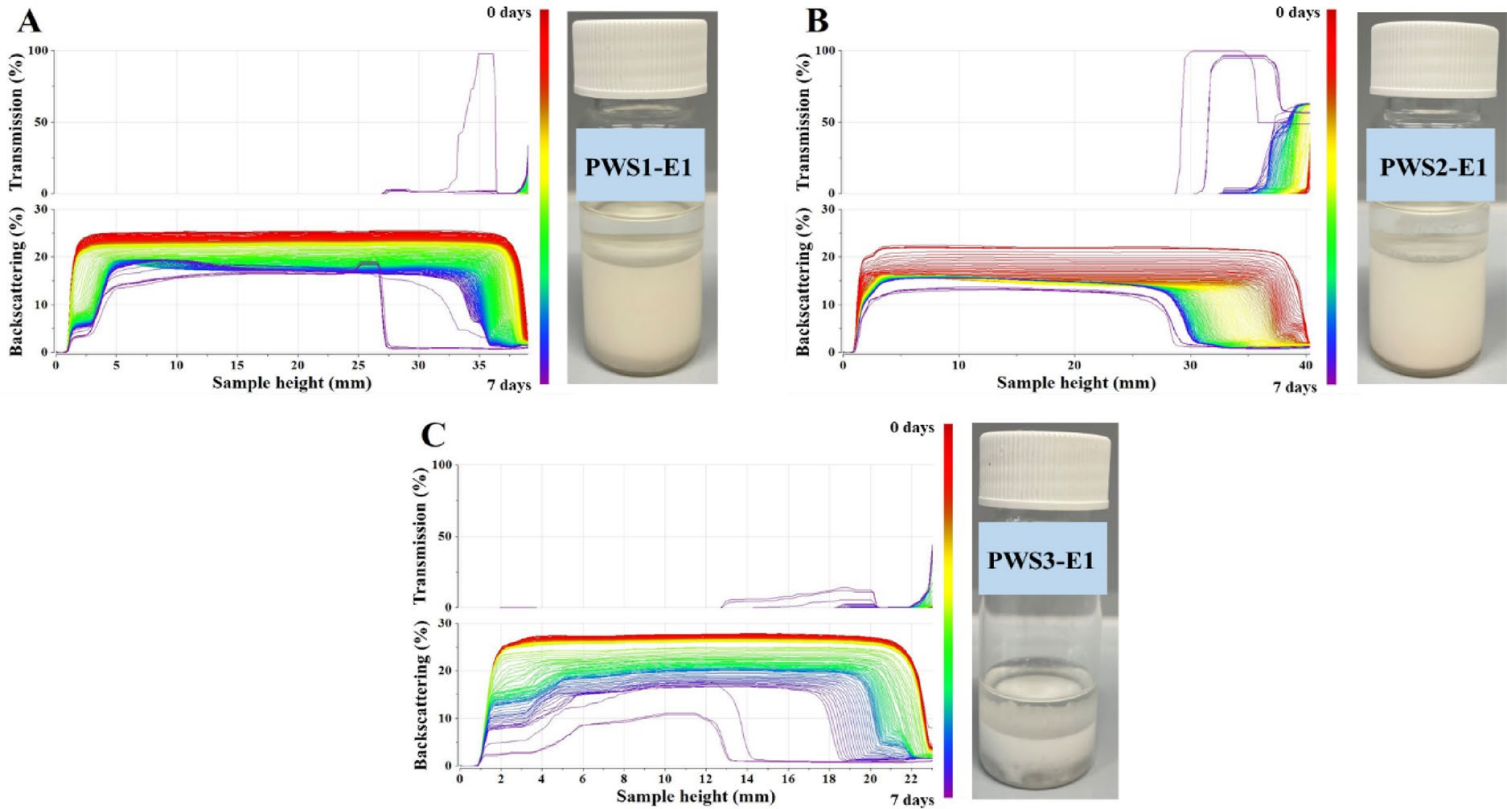
Transmission and backscattering profiles of PWS1-E1 (**A**), PWS2-E1 (**B**), PWS3-E1 (**C**).

Figure 8 shows the transmission and backscattering profiles of Emulsions 1 formulated with PWS1, PWS2, or PWS3 as the emulsifier, at an oil-to-water ration of 50:50% w/w. Phase separation of Emulsion 1 was observed after 2 days with PWS1, after 10 h with PWS2, and after 1.5 h with PWS3, as indicated in the transmission profiles.

These results clearly demonstrate the influence of emulsifier composition on emulsion stability. PWS1, modified with trimethoxysilane, eugenol, and octane in a 1:4:3 molar ratio, exhibited the highest stability, delaying phase separation for up to 48 h. This improved performance is likely due to the optimized balance between hydrophilic and lipophilic segments in the emulsifier structure. The higher proportion of eugenol and octyl groups enhances interfacial activity and facilitates the formation of a more coherent and elastic interfacial film, effectively stabilizing the emulsion droplets. In contrast, PWS2, modified with the same components but in a 1:2:1 molar ratio, resulted in reduced emulsion stability. The lower content of eugenol and octyl groups likely led to a less efficient interfacial film, weakening droplet stabilization and accelerating separation. The least stable system was observed with PWS3, which was modified only with eugenol and octane in a 3:1 molar ratio, lacking the trimethoxysilane component. The absence of trimethoxysilane likely reduced the emulsifier’s ability to anchor effectively at the oil–water interface and form hydrogen bonds or covalent interactions, resulting in rapid destabilization and phase separation. These findings underscore the importance of precise emulsifier design and component ratios in tailoring emulsion stability.

**Fig. 9:**
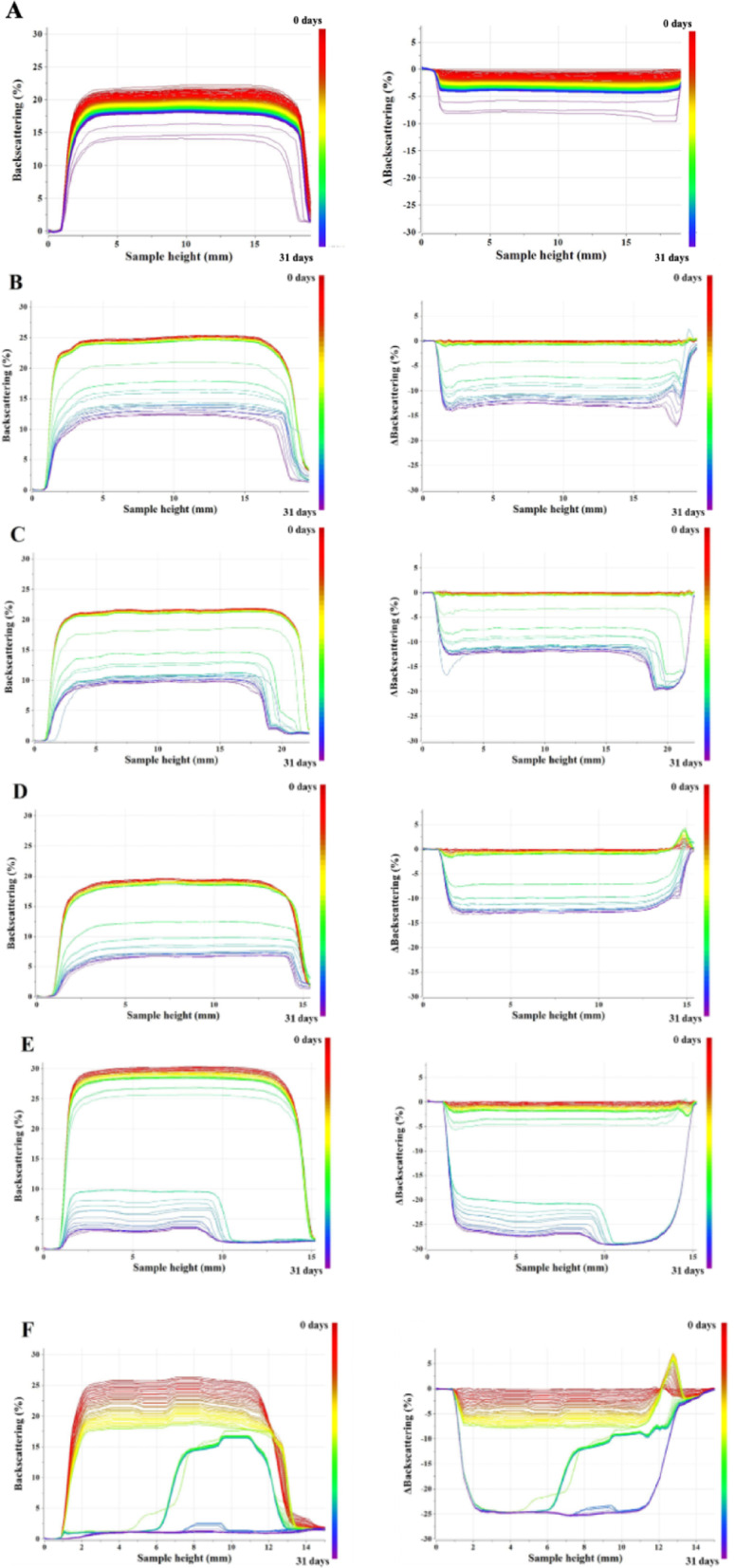
Backscattering profiles of PWS1-E2 (**A**), PWS1-E3 (**B**), PWS2-E2 (**C**), PWS2-E3 (**D**), PWS3-E2 (**E**), PWS3-E3 (**F**).

In turn, for Emulsion 2 and Emulsion 3 the results of stability studies were shown as backscattering profiles in the two following models: no-reference, which presents changes with respect to the first measurement (Fig. 9). The measurements were conducted for one month. Backscattering signals of emulsions obtained were changing along the whole sample height. It means that in these emulsions, flocculation (aggregation) of droplets occurred. The smallest difference between the initial and final measurements was observed for PWS1-E2, indicating that it was the most stable emulsion obtained.

In the case of emulsions with a water-to-oil phase ratio of 70:30% w/w and 80:20% w/w, formulations containing PWS1 also demonstrated superior stability compared to those containing PWS2 or PWS3. This enhanced stability can be attributed to the optimized composition of PWS1, which includes trimethoxysilane, eugenol, and octane in a molar ratio of 1:4:3. It is suggested, that the relatively high content of octane provides strong lipophilic interaction with the oil phase and promotes the formation of a flexible, cohesive interfacial film around dispersed oil droplets. Literature evidence shows that octane contributes to lowering interfacial tension and improving packing thereby promoting the formation of a flexible yet cohesive film that resists droplet coalescence^31^. Previous interfacial rheology studies have demonstrated that the identity of the oil species directly affects the viscoelastic properties of the interfacial film, with medium-chain alkanes such as octane penetrating into the surfactant tail region and thereby altering film structure and elasticity^31–34^. Neutron and X-ray reflectivity measurements further support the notion that short and medium alkanes can co-adsorb into surfactant monolayers, lowering interfacial tension and producing films that are both more flexible and cohesive^31,34^. Within the hydrophilic–lipophilic deviation (HLD) framework, octane has an equivalent alkane carbon number (EACN) of ~ 8, positioning it close to the “optimum formulation” range for balanced interfacial curvature and minimal interfacial tension^35^;^36^. Collectively, these findings provide a plausible mechanistic explanation for why formulations rich in octane display enhanced droplet stability: the hydrocarbon not only improves packing compatibility with surfactant chains, but also promotes the formation of a flexible, cohesive interfacial film that resists coalescence under stress.

This amphiphilic balance allows PWS1 to reduce interfacial tension more efficiently and to better prevent droplet coalescence and aggregation. Furthermore, the structural compatibility of PWS1 with both aqueous and oil phases results in a more robust stabilization of the dispersed system, even under conditions of limited oil content. These findings suggest that the superior emulsifying capacity of PWS1 in water-continuous systems is a direct consequence of its well-balanced hydrophilic–lipophilic profile and its ability to form stable interfacial layers, which are critical for maintaining emulsion integrity over time.

The most significant changes in the backscattering signal were observed for sample PWS3-E3, with phase separation detected after just 2 h of storage. In the case of sample PWS3-E2, signs of physical instability were also observed, but only after 3 days. Droplets flocculation can act as a precursor to phase separation in colloidal and emulsion systems. During the flocculation process, suspended particles aggregate into larger clusters (flocs). If these flocs grow sufficiently large and dense, they can physically separate from the continuous phase, resulting in the macroscopic phase separation of the system. In this study, the emulsions stabilized with PWS3, a polysiloxane modified with eugenol and octane, at water-to-oil ratios of 70:30% w/w and 80:20% w/w were investigated. During the initial stages of analysis, flocculation was observed in both systems. Over time, this flocculation led to phase separation, which was evident after one month. Notably, the phase separation occurred more rapidly in the 80:20% w/w (water: oil) system compared to the 70:30% w/w system. This observation suggests that the higher water content in the 80:20 system facilitates faster aggregation and subsequent phase separation, likely due to reduced continuous aqueous phase volume and altered interfacial properties. These findings highlight the importance of composition and emulsifier chemistry in determining the stability and lifetime of emulsions, as droplet flocculation-induced phase separation can limit product shelf life. In contrast, in other samples (PWS1-E2, PWS1-E3, PWS2-E2, PWS2-E3), a gradual decrease in backscattering intensity was observed over time along the entire height of the sample, indicating the occurrence of flocculation that was reversable process. Based on the obtained results the backscattering destabilization rates (BS%/day) were calculated (Table 6). It refers to the rate of changes in backscattering (BS) intensity over time, typically expressed in percentage per day. It quantifies how quickly the emulsion is destabilizing, such as through creaming, sedimentation, flocculation, or coalescence. A lower rate (close to 0%/day) indicates a physically stable formulation over the measured period. It enables to compare formulations over time^37^.

Quantitative analysis of the breakdown rates (BS%/day) revealed clear differences in emulsifier performance with statistical validation provided by on-way ANOVA followed by post-hoc Tukey’s HSD testing. In the evaluation of emulsifying performance of multifunctional polysiloxanes in various colloidal systems (50:50, 70:30, and 80:20% w/w water-to-oil ratios), PWS1 consistently exhibited the lowest destabilization rate (BS%/day) in all cases thus conferred superior emulsion stability. These findings were the most pronounced in E2 and E3 systems, where the differences between emulsifiers were statistically highly significant (ANOVA *p* < 0.0001) (Table 6). In E1, emulsifier differences were also significant (*p* = 0.0010), although pairwise comparisons revealed no statistical differences between PWS1 and PWS2 (*p* = 0.2794, Table 7), suggesting similar stabilizing effects in this formulation. In Emulsion E2 and E3, the breakdown rate of PWS3 was significantly different from both PWS1 and PWS2, indicating a reduced ability to stabilize the oil-water interface under the conditions tested. These findings reinforce the critical impact of emulsifier selection on formulation stability and provide a statistically robust basis for comparing emulsion performance.

**Table 6:** Rate of changes in backscattering over time.

Emulsion	Rate (BS %/day)	ANOVA *p*-value
PWS1-E1	− 1.833 ± 0.143	0.0010
PWS2-E1	− 2.124 ± 0.157	0.0010
PWS3-E1	− 2.811 ± 0.333	0.0010
PWS1-E2	− 0.295 ± 0.013	< 0.0001
PWS2-E2	− 0.489 ± 0.058	< 0.0001
PWS3-E2	− 1.021 ± 0,077	< 0.0001
PWS1-E3	− 0.419 ± 0.047	< 0.0001
PWS2-E3	− 0.466 ± 0.060	< 0.0001
PWS3-E3	− 1.056 ± 0.070	< 0.0001

**Table 7:** Summary of Tukey pairwise comparisons.

Emulsions	Emulsifier 1	Emulsifier 2	*p*-value	Interpretation
E1	PWS1	PWS2	0.2794	No significant difference
	PWS1	PWS3	0.0042	Significant difference PWS3 breakdown faster
	PWS2	PWS3	0.0250	
E2	PWS1	PWS2	0.1247	No significant difference
	PWS1	PWS3	< 0.0001	Siginificant difference PWS3 breakdown faster
	PWS2	PWS3	< 0.0001	
E3	PWS1	PWS2	0.1141	No significant difference
	PWS1	PWS3	< 0.0001	Siginificant difference PWS3 breakdown faster

**Fig. 10:**
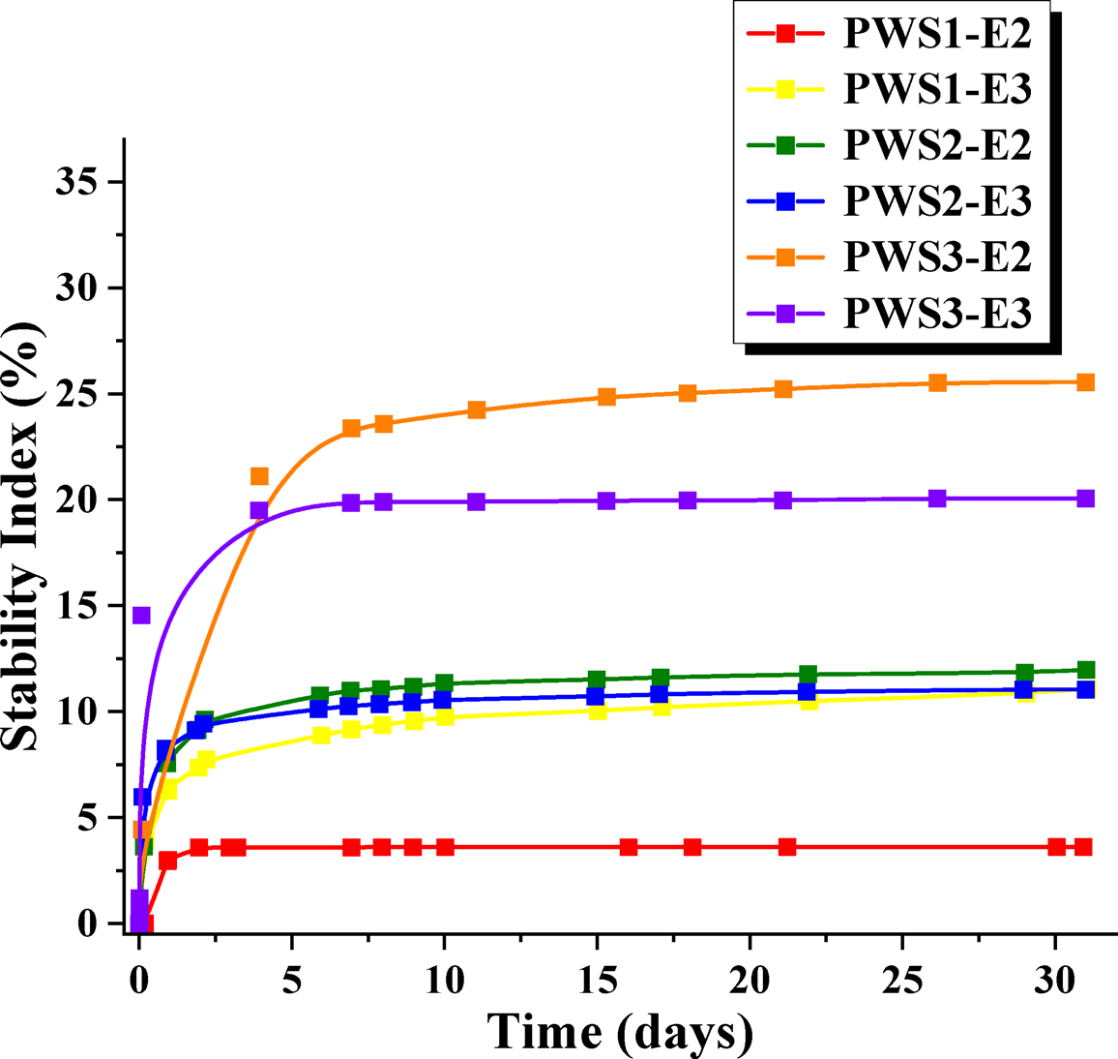
Stability indexes of PWS1-E2, PWS1-E3, PWS2-E2, PWS2-E3, PWS3-E2, PWS3-E3.

To better illustrate the changes occurring in the emulsions, the Stability Index (SI) was determined as a key parameter for evaluation the physical integrity of different emulsions. (Fig. 10). In literature, when stability is measured using a Turbiscan instrument, it is expressed as the Turbiscan Stability Index (TSI). Lower TSI values indicate higher stability of the tested formulation^38^. In our study, a Multiscan apparatus was applied, which calculates the Stability Index (SI) based on the same principle as the Turbiscan device. The SI is calculated according to the following formula:

$$\:SI=\sqrt{\frac{{\sum\:}_{i=1}^{n}{({x}_{i}-{x}_{BS})}^{2}}{n-1}}$$

where n is the number of measurement points, x_i_ is the backscattering value at a given point, x_BS_ is the mean backscattering over the measurement period.

Analysis of the Stability Index showed that emulsions containing the compound PWS3 were unstable—both in the case of formulations PWS3-E2 and PWS3-E3—as their Stability Index (SI) exceeded 10%^39^. According to the established criteria, samples with a SI value above 10% are classified as unstable. Consequently, emulsions PWS3-E2 and PWS3-E3 were excluded from further analysis. The highest stability was observed in the sample PWS1-E2, what is consistent with the results obtained from centrifugation test.

Microscopic images show the droplet sizes of the emulsions (PWS1-E2, PWS1-E3, PWS2-E2, and PWS2-E3) immediately after preparation and after one month of storage. Directly after preparation, all samples exhibited small, homogeneous droplet sizes. However, after one month of storage, an increase in droplet size was observed in all formulations, indicating the occurrence of droplet flocculation—a reversible process. The smallest droplet diameters were observed in the PWS1-E2 emulsion, which is consistent with the results obtained from centrifugation test and the multiple light scattering. After one month, the droplet sizes increased in the following order (from smallest to largest): PWS1-E2 < PWS1-E3 < PWS2-E2 < PWS2-E3.

**Fig. 11:**
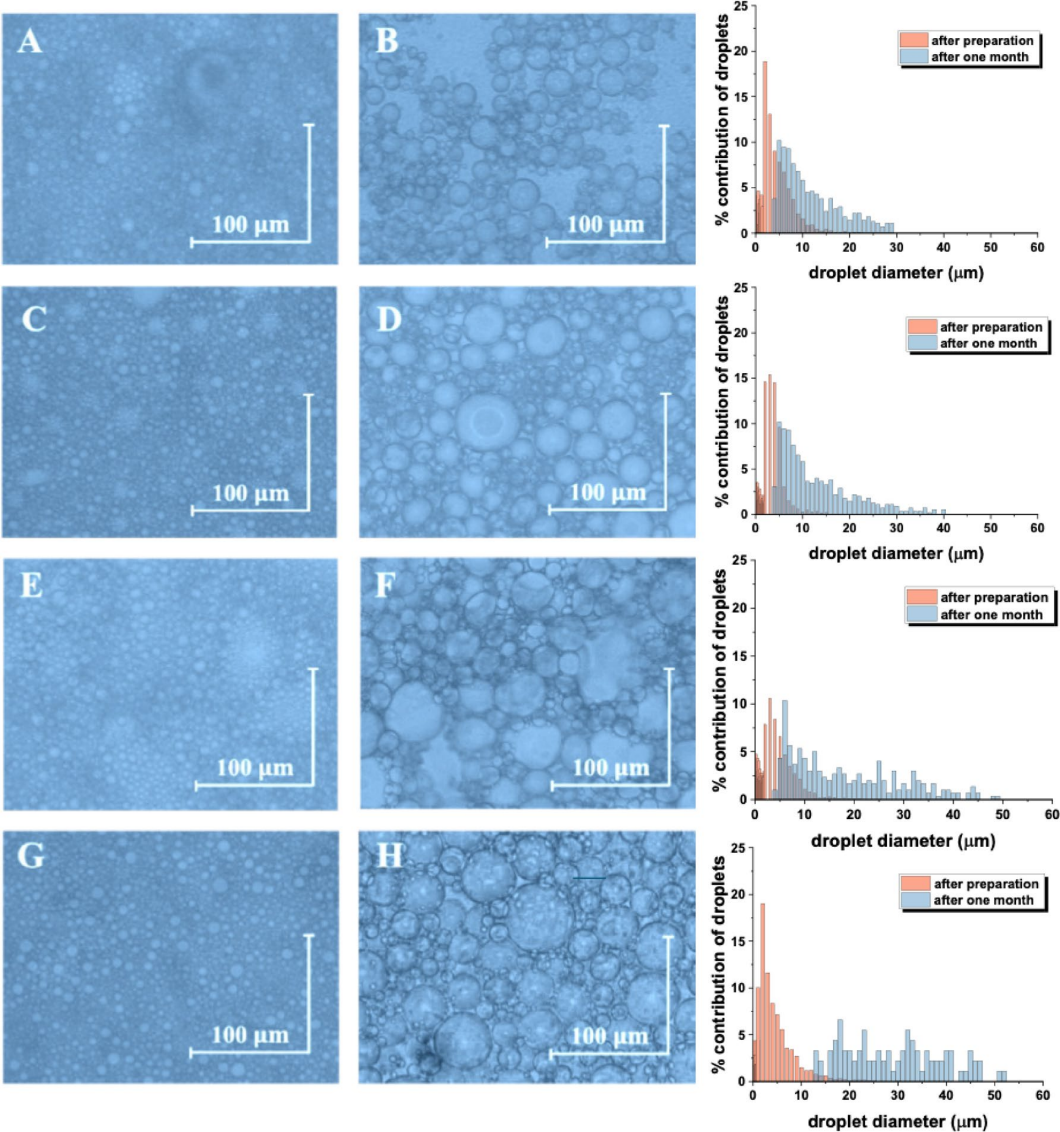
Microscope images of emulsions PWS1 E2 (**A**, **B**), PWS1 E3 (**C**, **D**), PWS2 E2 (**E**, **F**), PWS2 E3 (**G**, **H**) after preparation and after 1 month.

Microscopic evaluation of simple oil-in-water O/W and water-in-oil W/O emulsions stabilized with polysiloxane-based emulsifiers consistently demonstrates enhanced droplet-size stability compared with conventional surfactants. In W/O systems, emulsions prepared with cetyl PEG/PPG-10/1 dimethicone (also called polymethylsiloxane, EM90, ABIL) exhibit uniform droplets (ca.100 μm in diameter) with negligible coalescence, even after thermal stress, whereas emulsions stabilized with Span 80 showed pronounced droplet growth^40^. In O/W systems, emulsions stabilized with polyether-modified silicones displayed narrow droplet-size distributions and a robust interfacial lamellar structure that effectively suppressed coalescence^41^. Similarly, cosmetic W/O emulsions stabilized with cetyl dimethicone copolyol typically form 1–10 μm droplets that remain stable during storage, underscoring the role of polysiloxane emulsifiers in producing both thermally and kinetically stable dispersions^42,43^. In our studies, quantitative analysis of droplet diameters revealed clear differences between formulations. Emulsions PWS1-E2, PWS1-E3, PWS2-E2, PWS2-E3 (Fig, 11A, C, E, and G) exhibited significantly smaller average droplet sizes immediately after preparation compared with their respective counterparts stored for one month (Fig, 11B, D, F, and H; *p* < 0.0001). Post-hoc comparisons of mean droplet diameter further demonstrated that the magnitude of these differences varied among the predefined sample pairs. The smallest change was observed between PWS1-E2, with and average difference of 8.25 μm between freshly prepared emulsion (Fig. 11A) and that stored for one month (Fig. 11B, *p* = 0.0089). Larger differences were detected for PWS2-E2 (Fig. 11C, D mean difference = 10.99 μm, *p* < 0.0001), followed by PWS1-E3 (mean difference = 16.19 μm, *p* < 0.0001). The greatest increase was observed in PWS2-E3, where droplet diameters differed by 20.74 μm between preparation and one-month storage (Fig. 11G, H; *p* < 0.0001). These results indicate that although all paired samples showed significant increases in mean droplet size during storage, the extent of droplet growth was not uniform across formulations.

Our results show that the newly synthesized polysiloxane derivatives adsorb effectively at the oil–water interface and reduce interfacial tension, leading to stable emulsions. This behavior is consistent with previous reports on trisiloxane polyether surfactants, which are known to lower surface tension of water and form dense interfacial films that prevent droplet coalescence^26^. The multifunctional polysiloxanes proposed in this study differ from previously reported systems in which polysiloxane were modified solely with eugenol^19^. In the present work, two additional substitutes were introduced alongside eugenol, which significantly altered the physicochemical properties of the resulting polymers. This type of modified polysiloxanes may decrease the surface tension and show tendency to associate and self-assembly^44^.

The possibility to modify polysiloxane with three different functional groups opens several promising application areas. In the field of personal care formulations, such derivatives can function as efficient emulsifiers while simultaneously providing antimicrobial and antioxidant properties through eugenol, which has been widely reported to possess strong bioactivity^45,46^. The findings of our studies have practical implications for emulsion formulation, particularly in industries where long-term stability is critical, such as pharmaceuticals and cosmetics. The amphiphilic balance of polysiloxane derivatives also suggests potential in biomedical formulations and industrial additives, where surface activity, antimicrobial action, and dual compatibility with hydrophobic and polar phases are highly desirable. In addtion, trimethoxysilane substituents may enhance adhesion in coatings. Finally, in agrochemical formulations the modified polysiloxane could reduce surface tension and thereby improve pesticide spreading and foliar uptake^47^. These findings highlight the broad versatility of multifunctional polysiloxanes and their potential as a new platform for designing tailored emulsifiers across diverse industrial sectors.

## Conclusions

This study reports the synthesis, characterization, and application of novel multifunctional polysiloxanes (MPS) as advanced emulsifiers in colloidal systems. Three modified polysiloxanes (PWS1–PWS3) were obtained via hydrosilylation reactions, incorporating octane, trimethoxysilane, and eugenol in varying ratios to tailor their amphiphilic properties. Comprehensive characterization using NMR, FT-IR, TGA, and contact angle measurements confirmed the successful functionalization and thermal stability of the compounds within the range required for raw materials used in topical formulations. Key findings revealed that the modified polysiloxane synthesized using the balanced ratio of vinyltrimethoxysilane, eugenol, and octene (1:4:3), exhibited superior emulsion stability, attributed to enhanced interfacial interactions, including hydrogen bonding. In contrast, PWS3, lacking TMS, showed the lowest stability, highlighting the critical role of trimethoxysilane in emulsion stabilization. Emulsion stability was assessed via centrifugation tests, multiple light scattering, and optical microscopy, with PWS1-based emulsions demonstrating the highest resistance to phase separation. From the perspective of future research, this type of emulsifier may have potential applications in cosmetics (e.g., creams), pharmaceuticals (e.g., drug delivery systems), and industrial emulsions. In summary, it was shown that multifunctional polysiloxane systems can be precisely engineered for specific functional properties and can effectively address challenges related to colloidal stability.

## Data Availability

Correspondence and requests for materials should be addressed to Anna Olejnik.
